# Evolution of the pair rule gene network: Insights from a centipede^☆^

**DOI:** 10.1016/j.ydbio.2013.06.017

**Published:** 2013-10-01

**Authors:** Jack Green, Michael Akam

**Affiliations:** Laboratory for Development and Evolution, Department of Zoology, University of Cambridge, Downing Street, Cambridge CB23EJ, UK

**Keywords:** Segmentation, Pair rule gene, Pattern formation, Arthropod, Chilopod, Evolution

## Abstract

Comparative studies have examined the expression and function of homologues of the *Drosophila melanogaster* pair rule and segment polarity genes in a range of arthropods. The segment polarity gene homologues have a conserved role in the specification of the parasegment boundary, but the degree of conservation of the upstream patterning genes has proved more variable. Using genomic resources we identify a complete set of pair rule gene homologues from the centipede *Strigamia maritima*, and document a detailed time series of expression during trunk segmentation. We find supportive evidence for a conserved hierarchical organisation of the pair rule genes, with a division into early- and late-activated genes which parallels the functional division into primary and secondary pair rule genes described in insects. We confirm that the relative expression of *sloppy-paired* and *paired* with respect to *wingless* and *engrailed* at the parasegment boundary is conserved between myriapods and insects; suggesting that functional interactions between these genes might be an ancient feature of arthropod segment patterning. However, we find that the relative expression of a number of the primary pair rule genes is divergent between myriapods and insects. This corroborates suggestions that the evolution of upper tiers in the segmentation gene network is more flexible. Finally, we find that the expression of the *Strigamia* pair rule genes in periodic patterns is restricted to the ectoderm. This suggests that any direct role of these genes in segmentation is restricted to this germ layer, and that mesoderm segmentation is either dependent on the ectoderm, or occurs through an independent mechanism.

## Highlights

• We use genomic resources to identify 17 *Strigamia* pair rule gene homologues.
• Sixteen of these are expressed in periodic patterns before the appearance of morphological segments.
• We allocate genes to primary and secondary tiers in the hierarchy based on detailed time series data.
• Expression of *pax3/7* and *slp* homologues is conserved at the parasegment boundary across arthropods.
• Segmental expression is restricted to ectoderm, suggesting mesoderm segmentation is secondary.

## Introduction

The genetic dissection of the mechanism of segmentation in the fruit fly, *Drosophila melanogaster*, has laid the foundation for a growing body of comparative research on other arthropods (Nusslein-Volhard and Wieschaus, 1980; Peel et al., 2005). The *Drosophila* work identified a number of genes involved in segment pattern formation, and divided them into four functional categories based on their mutant phenotypes. These categories are maternal factors, gap genes, pair rule genes and segment polarity genes. Work over the years has pieced together the hierarchy of interactions between these genes, and shown how this hierarchy is capable of reproducibly generating precise patterns (Akam, 1987; Jaeger et al., 2004; Pankratz and Jaeckle, 1993).

Homologues for most of the *Drosophila* segmentation genes can be identified throughout the arthropods. The key segment polarity genes *wingless, engrailed, hedgehog* and *Cubitus interruptus* show highly conserved expression at the parasegment boundary in a range of arthropod species, including chelicerates and myriapods, as well as insects (Damen, 2007; Farzana and Brown, 2008). Thus, the parasegment is likely to be an ancient feature of arthropod segmentation. However, the extent of conservation in the gene regulatory network that acts upstream of the segment polarity genes is not yet clear (Peel et al., 2005).

Besides *Drosophila*, there are two other insects for which extensive functional data are available on the role of pair rule gene homologues: the red flour beetle *Tribolium castaneum* (Choe and Brown, 2007, 2009; Choe et al., 2006) and the honeybee *Apis mellifera* (Wilson and Dearden, 2012). In the honeybee, transcripts for a number of the pair rule gene homologues are deposited into the oocyte and have acquired novel roles in early developmental patterning, which obscure any later roles in segmentation in RNAi experiments (Wilson and Dearden, 2012). Therefore, the main work able to address the role of these genes in segment pattern formation is in *Tribolium*. There are a number of differences in the roles of pair rule genes between *Tribolium* and *Drosophila*. For example, the beetle homologues of the pair rule genes *hairy*, *odd-paired* (*opa*) and *fushi-tarazu* (*ftz*) appear to have no essential role in *Tribolium* trunk segmentation, or at least give no detectable RNAi phenotype (Aranda et al., 2008; Choe et al., 2006).

One aspect of segment patterning that is largely conserved between *Tribolium* and *Drosophila* is the division of the pair rule genes into primary and secondary tiers, based on their functional position in the hierarchical cascade. Primary pair rule genes are upstream in the cascade and are regulated by other factors (maternal coordinate factors and gap genes), whereas the secondary pair rule genes are downstream in the cascade and regulated by the primary pair rule genes. In *Drosophila*, *even-skipped* (*eve*), *runt* and *hairy* are considered the primary pair rule genes; *sloppy-paired* (s*lp*) and *paired* (*prd*) are considered the secondary pair rule genes. *ftz* and *odd-skipped* (*odd*) were originally considered to be secondary, but a more recent and thorough analysis of their cis-regulatory architecture has shown that in some respects they qualify as primary pair rule genes (Schroeder et al., 2011). In *Tribolium*, *eve*, *runt,* and *odd* are found to be primary pair rule genes, and *slp* and *prd* to be secondary pair rule genes. Thus, not only do many of the *Tribolium* pair rule gene homologues play a role in segmentation, they are also found to occupy similar levels in the gene regulatory hierarchy. (Choe et al., 2006).

In non-insect arthropods there is indirect evidence that the functional division of the pair rule network into primary and secondary levels is conserved. Importantly, the timing of expression of the pair rule genes in segment patterning reflects the functional division of the hierarchy. That is to say, the alternate expression of the primary pair rule genes is established first in the process, and the periodic patterning of the secondary pair rule genes appears afterwards. Therefore in arthropods where functional tools are not available, indirect evidence for the functional division can still be obtained from the temporal order in which the genes are expressed during segment pattern formation. This has been done for spider (Damen et al., 2005) and millipede segmentation (Janssen et al., 2011). A consistent finding across studies is the early expression, and where tested, upstream function of *eve*, *runt* and *odd* homologues during segment patterning; and the late expression, and where tested, downstream function of *prd* and *slp* homologues. This suggests that the functional division between primary and secondary pair rule genes, and at least some of the genes that occupy these categories, may be a conserved feature of arthropod segmentation.

A second aspect of segmental patterning conserved between *Tribolium* and *Drosophila* concerns the role of the secondary pair rule genes, *prd* and *slp*, in the regulation of the key segment polarity genes, *wingless* (*wg*) and *engrailed* (*en*) (Choe and Brown, 2009). In both insects, the *prd* homologue is expressed in both *wg*- and *en*-positive cells, and thus overlaps the parasegment boundary, whereas the *slp* homologue is restricted to *wg*-positive cells. The same relationship is observed between these four genes in the pill millipede *Glomeris marginata* (Janssen et al., 2011), and the available data suggests that it holds for a *pax3/7* homologue and *en* in the grasshopper *Schistocerca americana* (Davis et al., 2001). This striking conservation of relative expression suggests an ancient origin for the regulatory module in which these four genes act, even accepting that conserved transcriptional output cannot be taken to imply that the underlying transcriptional networks are also conserved (Ludwig et al., 2000; Romano and Wray, 2003).

We have tested whether these features of the pair rule gene network are also conserved in another ancient branch of the myriapods – the Chilopoda. Our study species is the geophilomorph centipede, *Strigamia maritima*. Previous work on *Strigamia* has characterised a number of the major features of segmentation (Brena and Akam, 2012; Chipman et al., 2004b; Kettle et al., 2003). At the time of germ band formation, a large population of progenitor cells forms a disc of unsegmented tissue at the posterior of the embryo. As the germ band elongates, this posterior disc narrows and segments emerge from it sequentially in anteroposterior order. Because segments are added in a temporal sequence, the progression of segment patterning can be visualised in each embryo as a sequence from posterior to anterior.

Expression of some of the pair rule gene homologues has already been examined in *Strigamia* – two *even-skipped* homologues (*Sm-eve1* and *Sm-eve2*); one member of the *odd-skipped* gene family (*Sm-odr1*); and two members of the *hairy*/*deadpan* family (originally named *Sm-hes1* and *Sm*-*hes4*) (Chipman and Akam, 2008; Chipman et al., 2004a). This work has shown that there are two phases of patterning during the major stage of trunk segmentation. In the first phase, dynamic patterns of gene expression resolve to a series of concentric rings in the posterior disc, defining a double segment repeat. In the second phase, the double segment pre-pattern resolves to a single segment repeat by the splitting and/or intercalation of expression domains. The region where this segmental resolution occurs, in between the posterior disc and the segmented germ band, is called the transition zone. The domain of dynamic expression is restricted to a population of cells surrounding, but largely anterior to, the proctodeum and lying within the first resolved ring of expression. We refer to this territory as the peri-proctodeal region.

We set out to test four hypotheses about the degree of conservation of the pair rule gene homologues in segment pattern formation. First, are the pair rule genes in *Strigamia* hierarchically organised into primary and secondary tiers, as has been described in other arthropods? Second, are the relative expression domains of key genes conserved during the specification of the parasegment boundary? In particular, is the spatial registration of *slp* and *pax3/7* homologues conserved in relation to the abutting *wg*- and *en*-expressing cells? Third, is there evidence from the relative expression of *eve*, *runt*, *odd* and *hairy* homologues that the upstream tiers of segment patterning are more divergent? Fourth, is the expression of the *Strigamia* pair rule genes restricted to the ectoderm germ layer? And if not, are any of these genes expressed in patterns that suggest an early role in mesoderm segmentation? We have addressed these hypotheses by examining the spatial and temporal dynamics of expression of a complete set of *Strigamia* pair rule homologues during segment patterning.

## Materials and methods

### Embryo collection, fixation and staging

Embryos were collected in the field from a population near Brora, Scotland and fixed as described previously (Chipman et al., 2004b). Embryos were staged by counting the total number of morphologically visible leg-bearing segments, either in whole embryos or in flat-mount preparations.

### Gene identification and cloning

A genome and adult and embryonic transcriptomes have recently been assembled for *Strigamia maritima* (genome release Smar_1.0 http://www.ncbi.nlm.nih.gov/assembly/322118/). Pair rule gene homologues were identified by similarity searches against these resources, and orthology of the genes was confirmed by reciprocal similarity searches. To clone genes, gene-specific primers were designed against the genomic or transcriptomic sequence, and products amplified by standard PCR from embryonic cDNA. Genes were cloned into a pGEM-T Easy vector (Promega). The clones of *ftz* and *twist* were a gift of C. Brena, the clones of *slp* and *opa1* were a gift of V. Hunnekuhl and the clone of *wg* was a gift of L. Hayden. An annotated gene set for *Strigamia* is provided at EnsemblMetazoa (http://metazoa.ensembl.org/Strigamia_maritima/Info/Index). The Ensembl IDs of the pair rule and segment polarity gene homologues examined in this paper are provided in Supplementary Table 1.

### Phylogenetics

Phylogenetic analysis of the genes was carried out using Phylemon2 (Sanchez et al., 2011). The multiple sequence alignment was performed on protein sequences using MUSCLE (Edgar, 2004). The gene trees were built by maximum likelihood analysis in PhyML (Guindon and Gascuel, 2003) using the LG substitution model with 100 bootstrap replicates. We used empirical frequencies, had four substitution rate categories and a proportion of invariant sites, and estimated the gamma distribution parameter. Trees were visualised in the programme FigTree v1.3.1 (Rambaut, 2009). For clarity, any nodes with less than 70/100 bootstrap support were collapsed to polytomies using the programme TreeCollapserCL v3.2 (Hodcroft, 2012).

### Whole mount in situ hybridisation

Single and double colorimetric *in situ* hybridisation reactions were carried out on whole mount embryos as described previously (Chipman et al., 2004b; Chipman and Stollewerk, 2006). After staining, the embryo was dissected away from the yolk with fine tweezers and flat-mounted under a cover slip on a microscope slide. Bright field or DIC images of embryos were taken on a Zeiss Axiophot compound microscope with a Leica DFC 300 FX camera. Fluorescent images and optical sections were taken using a Leica TCS SP5 confocal microscope. The photos were adjusted using Adobe Photoshop (version CS5).

### A note on terminology

The term “pair rule” has been used in three different ways in the literature. The original sense of the term was a functional categorisation based on the loss-of-function mutant phenotype (Nusslein-Volhard and Wieschaus, 1980). Second, the term was used to describe genes expressed in a double segment pattern, even if the loss-of-function phenotype was not consistent with the classical pair rule phenotype (Choe et al., 2006). Third, the term has been used as a convenient ‘category-level name’ for the set of genes in other species that are orthologous (one-to-one or one-to-several) to the set of genes historically identified in *Drosophila* under that name (Damen et al., 2005; Janssen et al., 2011). This third use only implies homology of the genes themselves between the taxa of interest, and explicitly makes *no* statement on homology at the level of expression or function of the genes. In this paper, we use the term “pair rule gene” in this third sense.

## Results

### A complete set of pair rule gene homologues identified and cloned in *Strigamia*

**Supplementary Fig. S1:**
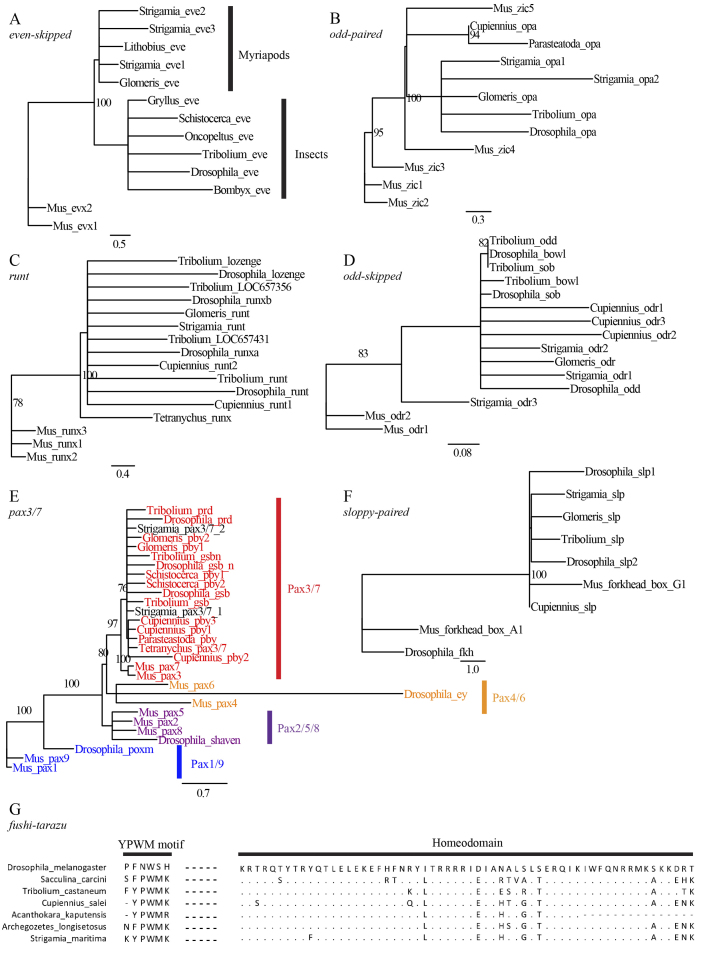
Phylogenetic analysis of *Strigamia* pair rule gene homologues. Phylogenetic trees for homologues from various arthropods and the mouse of (A) *even-skipped*, (B) *odd-paired*, (C) *runt*, (D) *odd-skipped*, (E) *paired* and related pax genes, (F) *sloppy-paired*. (G) Protein alignment of *fushi-tarazu* orthologues from various arthropods. Values at nodes are bootstrap support values (out of 100 bootstrap replicates). Nodes with less than 70/100 support are collapsed to polytomies. Abbreviations for genes: eve, *even-skipped*; evx, *even-skipped homeobox*; opa, *odd-paired*; zic, *zinc finger protein*; odd, *odd-skipped*; odr, *odd-skipped-related*; bowl, *brother of odd with entrails limited*; sob, *sister of odd and bowl*; prd, *paired*; gsb, *gooseberry*; gsb-n, *gooseberry-neuro*; pby, *pairberry*; pax, *paired box*; ey, *eyeless*; poxm, *pox-meso*; slp, *sloppy-paired*; fkh, *forkhead*. Abbreviations for animals: Bombyx, *Bombyx mori* (silkmoth); Cupiennius, *Cupiennius salei* (spider); Drosophila, *Drosophila melanogaster* (fly); Glomeris, *Glomeris marginata* (millipede); Gryllus, *Gryllus bimaculatus* (cricket); Lithobius, *Lithobius atkinsoni* (lithobiomorph centipede); Mus, *Mus musculus* (mouse); Oncopeltus, *Oncopeltus fasciatus* (milkweed bug); Parasteatoda, *Parasteatoda tepidariorum* (spider); Schistocerca, *Schistocerca americana* (grasshopper); Strigamia, *Strigamia maritima* (centipede); Tetranychus, *Tetranychus urticae* (mite); Tribolium, *Tribolium castaneum* (beetle).

Based on current genomic and transcriptomic resources, we believe that we have identified and cloned all the *Strigamia* homologues of the eight canonical *Drosophila* pair rule genes. In total 17 genes are identified and characterised: one homologue each of *runt* (*Sm-run*), *sloppy-paired* (*Sm-slp*) and *fushi-tarazu* (*Sm-ftz*); two homologues of the *paired/gooseberry/gooseberry-neuro* class of genes (*Sm-pax3/7-1* and *Sm-pax3/7-2*); two homologues of *odd-paired* (*Sm-opa1* and *Sm-opa2*); three homologues each of *even-skipped* (*Sm-eve1, Sm-eve2* and *Sm-eve3*) and *odd-skipped* (*Sm-odr1*, *Sm-odr2*, *Sm-odr3*); and four *hairy/deadpan* homologues (*Sm-h1*, *Sm-h2*, *Sm-h3, Sm-h4*). Phylogenetic analysis confirms that there is no ambiguity about the many-to-many orthology of the genes examined, and suggests that, with the exception of *hairy*, where there are multiple gene family members in *Strigamia*, these are the result of lineage-specific duplications within the arthropods (Fig. S1). In general, it is not possible to define one-to-one orthologous relationships between the genes. For example, the myriapod family of *eve* genes form a separate clade from the insect family, but relationships within the myriapod clade are otherwise unresolved (Fig. S1A).

Phylogenetic analysis identifies *opa2* as a fast-evolving, divergent sequence (Fig. S1B). *opa2* appears not to be expressed in embryos – transcripts are not identified in the embryonic transcriptome, and the gene failed to amplify from embryonic cDNA (results not shown). However, reads for this gene are present in the adult transcriptome, showing that *opa2* is expressed in adult tissue. The high divergence of the sequence is consistent with the gene adopting a specialised or novel role in adults.

There are multiple *runt*-related genes in other arthropods, for example *runt*, *lozenge*, *runxa* and *runxb* in *Drosophila* (Bao and Friedrich, 2008). Analysis of the amino acid sequence of the single *Strigamia runt* homologue confirms its orthology to the arthropod *runt* genes. It contains the canonical *runt* DNA-binding domain and WRPY motif (results not shown). However, there is no good evidence for a one-to-one orthologous relationship between the *Strigamia runt* gene and any other arthropod *runt*-related genes (Fig. S1C).

Three *odd* homologues are identified in *Strigamia* by the presence of conserved zinc-finger domains and their high similarity to *odd* family members from other arthropods (results not shown). Phylogenetic analysis shows no strong evidence for any one-to-one orthology between the *Strigamia* genes and the insect *odd*/*sob*/*bowl* class of genes, nor between them and any chelicerate *odd* family members (Fig. S1D). We therefore refer to these genes as *odd-skipped-related1*, *2* and *3* (*odr1*, *odr2* and *odr3*).

Two putative homologues of the *Drosophila prd* gene are identified in *Strigamia*. The *Drosophila* pair rule gene *prd*, and the closely related *gooseberry* genes, are the *Drosophila* Pax3/7 family members. There are four major families of Pax genes in bilaterians – Pax1/9, Pax2/5/8, Pax4/6 and Pax3/7 (Balczarek et al., 1997). Phylogenetic analysis shows that the two *Strigamia* homologues form a well-supported Pax3/7 clade to the exclusion of the other Pax families (Fig. S1E). However, orthology relationships within the clade are not well resolved. We therefore refer to these genes in *Strigamia* as *pax3/7-1* and *pax3/7-2*.

*slp* is a Fox family transcription factor. There are 17 subclasses in the Fox family (Mazet et al., 2003). In *Drosophila melanogaster*, there are three members of the FoxG subclass, *slp1*, *slp2* and *forkhead domain 19B* (Lee and Frasch, 2004; Mazet et al., 2003). Phylogenetic analysis shows that there is only one member of the FoxG subclass in *Strigamia*, which we refer to as *slp* (Fig. S1F).

The *Strigamia* orthologue of *ftz* is identified by conserved residues in the homeodomain (Fig. S1G) and by its genomic position in a conserved Hox cluster – it lies between the orthologues of S*ex combs reduced* and *Antennapedia* (genome release Smar_1.0).

Phylogenetic analysis of *hairy*, *deadpan*, *enhancer of split* and other basic helix-loop-helix (bHLH) genes was carried out by L. Duncan and P. Dearden as part of the *Strigamia maritima* genome annotation (unpublished data). It identified four members of a *hairy*/*deadpan* clade in *Strigamia.* These genes are distinct from genes in the *enhancer of split* clade, and from other bHLH genes. Therefore we refer to these as *hairy1*, *2*, *3* and 4 (*h1*, *h2, h3* and *h4*). *h1*, *h2* and *h3* are linked in a 30 kb genomic cluster, whereas *h4* is on a separate genomic scaffold. Four *hairy*/*enhancer of split* (HES) related genes were reported previously (Chipman and Akam, 2008). Two of these, both isolated as cDNA clones, correspond to two of the genes defined in the genome annotation: *hes1* becomes *h4*, and *hes4* becomes *h3*. The other two (*hes2* and *hes3*) were previously recovered only as short fragments from a PCR screen. They show no close match at the nucleotide level to any sequence in the *Strigamia* genome or transcriptome assemblies. They may be derived from contaminating PCR fragments in the original study.

### A hierarchy of primary and secondary pair rule genes in *Strigamia*

**Supplementary Fig. S2:**
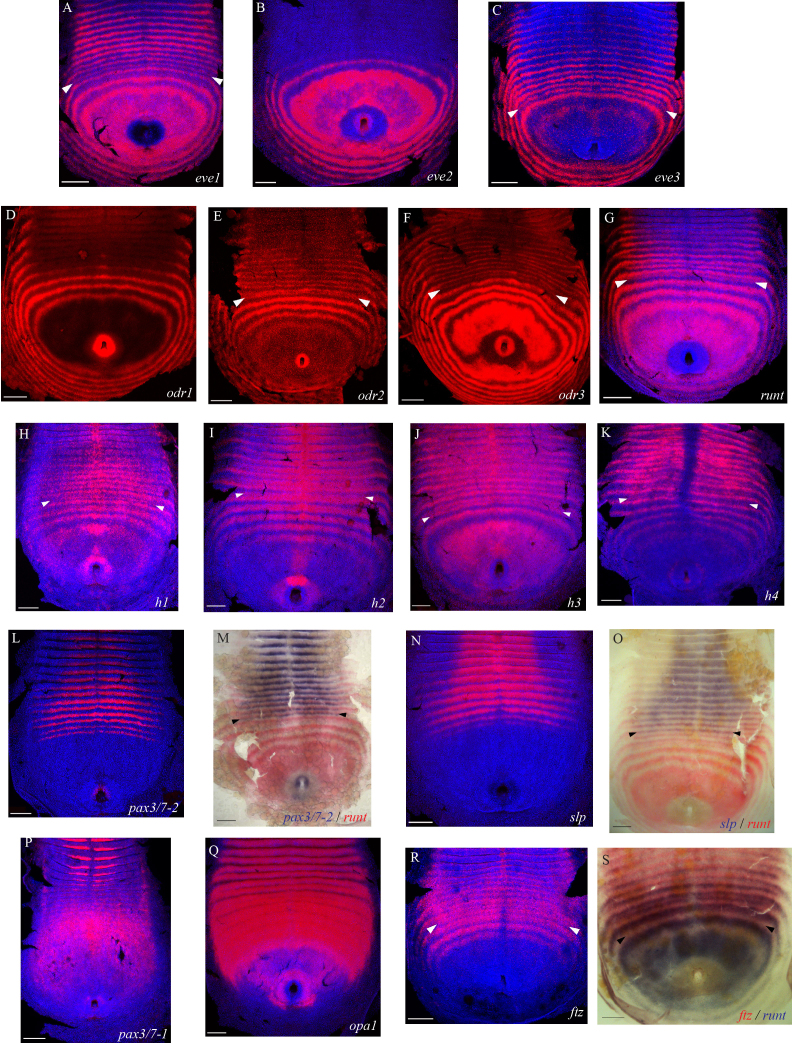
Expression of pair rule gene homologues during *Strigamia* trunk segmentation. (A) Expression of *eve1* in the posterior disc and germ band of a stage 4 *Strigamia* embryo with 21 leg-bearing segments. (B) *eve2*, 17 lbs. (C) *eve3*, 21 lbs. (D) *odr1*, 25 lbs. (E) *odr2*, 18 lbs. (F) *odr3*, 18 lbs. (G) *runt*, 23 lbs. (H) *h1*, 14 lbs. (I), *h2*, 17 lbs. (J) *h3*, 23 lbs. (K) *h4*, 16 lbs. (L) *pax3/7-2*, 28 lbs. (M) Double stain for *pax3/7-2* (blue) and *runt* (red), 20 lbs. The first stripe of *pax3/7-2* appears overlapping with the posterior cell rows of the first intercalating *runt* stripe (black arrowheads). (N) *slp*, 33 lbs. (O) *slp* (blue) and *runt* (red), 19 lbs. The first stripe of *slp* appears overlapping with the first primary stripe of *runt*, after two secondary *runt* stripes have intercalated (black arrowheads). (P) *pax3/7-1*, 22 lbs. (Q) *opa1*, 25 lbs. (R) *ftz*, 18 lbs. (S) *ftz* (red) and *runt* (blue), 24 lbs. *ftz* transcripts first appear at the anterior margin of the peri-proctodeal region, anterior to the first demarcated ring of *runt* (black arrowheads). White arrowheads indicate the transition from double to single segment periodicity, through the intercalation of new stripes in between the primary stripes (A, C, E, F, G, H, J, K), or the splitting of broad, primary stripes into alternating long and short stripes (I, R). (A, B, C, G, H, I, J, K, L, N, P, Q, R) RNA is stained in red and nuclei are stained in blue. The expression pattern is overlain on the nuclear stain. (D, E, F) RNA expression is shown on its own, with no nuclear stain overlay. This is to better visualise the weaker, new stripes of expression that intercalate between the primary stripes, which are otherwise obscured against the background of the nuclear stain. Embryos are flat-mounted preparations. Anterior is to the top. Scale bar is 100 μm.

**Fig. 1:**
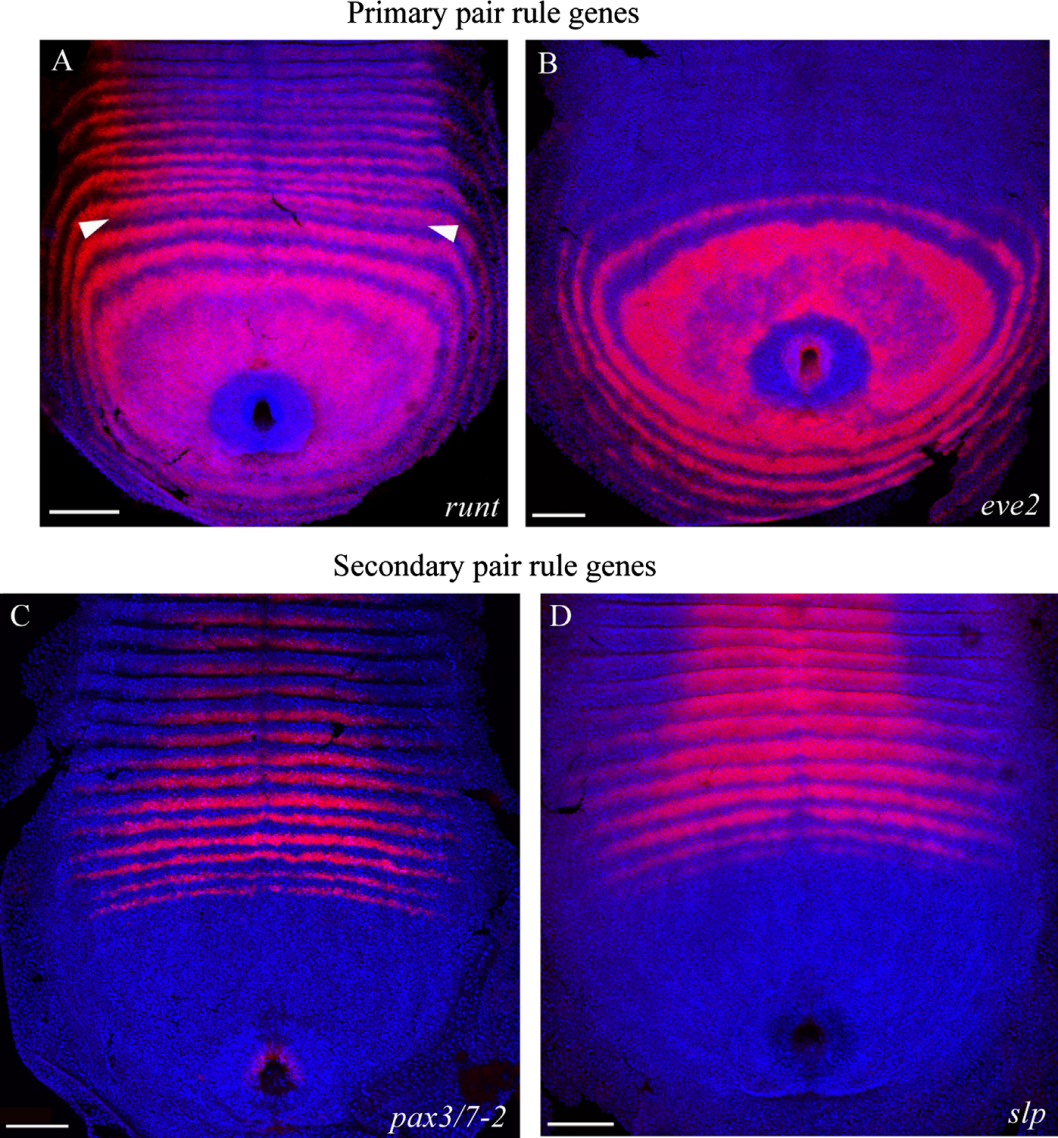
Examples of primary and secondary pair rule genes in *Strigamia.* (A) Expression of the primary pair rule gene *runt* in the posterior disc and segmenting germ band of a stage 4 *Strigamia* embryo with 23 leg-bearing segments (lbs). *runt* initiates as a dynamic domain in the peri-proctodeal region and resolves to a series of concentric rings at double segment periodicity. These broad stripes then resolve to a single segment repeat through the intercalation of a shorter stripe between two broad stripes (white arrowheads). (B) Expression of *eve2* at a similar stage, 17 lbs. *eve2* initiates in a similar pattern to *runt*, but is inactivated after one or two rings have resolved from the dynamic domain. It never undergoes resolution to a single segment periodicity. (C) Expression of the secondary pair rule gene *pax3/7–2*, 28 lbs. (D) Expression of *slp*, 33 lbs. *pax3/7-2* and *slp* initiate at a more anterior position in the germ band, and are only ever expressed at single segment periodicity. Anterior is to the top. Scale bar is 100 μm. For similar data on the complete set of primary and secondary pair rule genes, see Supplementary Fig. 2.

We report here the expression of 16 pair rule gene homologues during *Strigamia* trunk segmentation (Figs. 1 and S2). All the embryos examined are at stage 4.2 or early 4.3, during the major phase of segment addition; stage 4.2 embryos have between ≈14 and ≈27 leg-bearing segments (Brena and Akam, 2012). Note that our descriptions are restricted to the segmenting tissue of the embryo, including the peri-proctodeal territory, but they exclude the tissues of the proctodeum itself and the immediately adjacent tissue. These comprise a unique set of cell populations, including those associated with the future gut. They will be described in detail elsewhere.

With the exception of *opa2*, all of the genes studied are expressed in the tissue of the posterior disc and/or the forming germ band, before the appearance of morphological segments. Therefore, these genes may play some role in segment patterning. All of the genes are activated before the first expression of the segment polarity gene homologues *en* and *wg* (Fig. 3). This is consistent with a hierarchical role upstream of parasegment boundary specification.

From an analysis of the expression dynamics, there is good evidence that the *Strigamia* pair rule genes are hierarchically organised into primary and secondary tiers. A subset of the genes is activated early in the segmentation process and initiates at a double segment periodicity in the posterior disc (Figs. 1A, B and S2A–K). This set of genes includes all the *Strigamia* homologues of *eve*, *runt*, *odd* and *hairy*. The activation of these genes early in the progression of the pattern is consistent with them operating upstream in the regulatory network. We shall refer to them as primary pair rule genes. Most of these genes resolve to a single segment repeat by the intercalation or splitting of expression domains in the transition zone (Fig. 1A). However, two of them (*eve2* and *odr1*) are inactivated before this, and never make the transition to single segment periodicity (Figs. 1B and S2D).

In contrast, a second set of genes is first activated at a more anterior position in the germ band. These are *pax3/7-2* and *slp* (Fig. 1C, D). We designate these as secondary pair rule genes because their later activation in the development of the pattern is consistent with them occupying a downstream tier in the network. They show no evidence of being activated in a double segment pre-pattern. Rather, they initiate at single segment periodicity, coincident with or shortly after, the transition of the primary pair rule genes to a single segment repeat (Fig. S2M and O). Their expression never appears before the primary pair rule genes undergo this transition.

Therefore, there is clearly a division into two classes of genes that initiate at different periodicities and at different points in the progression of pattern formation. This is consistent with a hierarchical organisation of these gene classes in the regulatory network. It is striking that homologous genes occupy the same relative positions in the hierarchy as in other arthropods examined to date.

Three genes do not fit comfortably into either of these categories, *pax3/7-1*, *opa1* and *ftz* (Fig. S2P, Q and R). *pax3/7-1* is not expressed in the ectoderm before morphological segmentation, but is restricted to a subset of mesodermal cells (Fig. S2P and S4N). This is addressed in more detail in a following section.

Transcription of *opa1* initiates in the posterior disc and transcripts persist through the transition zone and into nascent morphological segments (Fig. S2Q). However, *opa1* expression shows no modulation in relation to the forming segment pattern; any appearance of modulation is actually caused by cell rearrangements during the folding in of furrows at morphological segmentation.

The expression of the *ftz* orthologue exhibits a double segment modulation. It initiates as broad double segment stripes at the anterior margin of the peri-proctodeal region. In the transition zone these resolve into alternating long and short segmental stripes. However, it does *not* show any dynamic expression in the peri-proctodeal region itself (Fig. S2R). This suggests that *ftz* is entrained by the upstream pair rule patterning system, but is not involved in the primary pattern generation.

### Map of relative expression domains for the *Strigamia* pair rule genes

**Supplementary Fig. S3:**
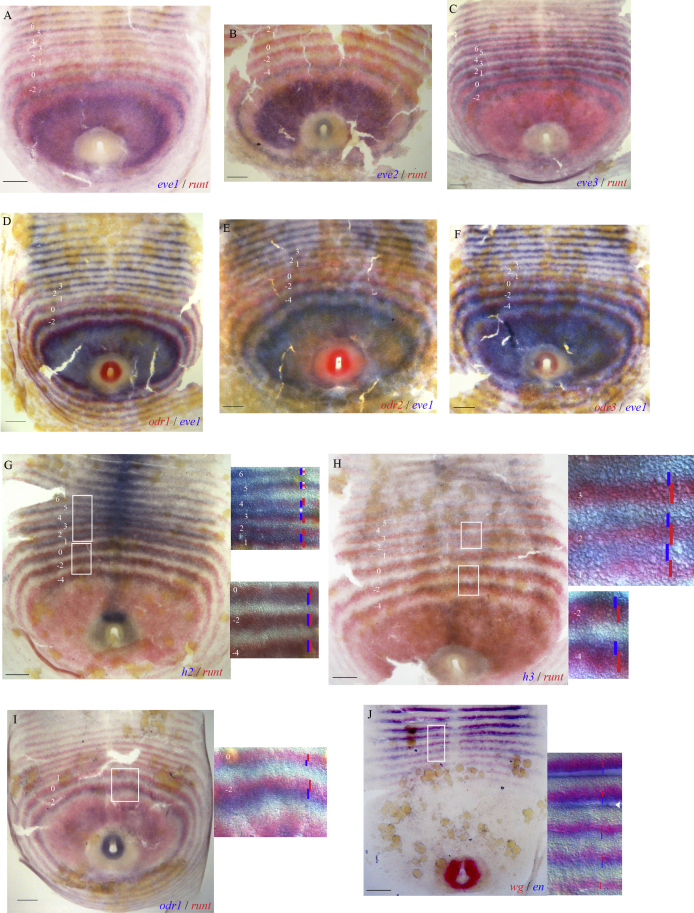
Registration of the pair rule genes during the progression of pattern formation. (A) Expression of *eve1* (blue) and *runt* (red) on a stage 4 embryo with 18 lbs. (B) *eve2* (blue) and *runt* (red), 19 lbs. (C) *eve3* (blue) and *runt* (red), 19 lbs. (D) *odr1* (red) and *eve1* (blue), 23 lbs. (E) *odr2* (red) and *eve1* (blue), 19 lbs. (F) *odr3* (red) and *eve1* (blue), 19 lbs. (G) *h2* (blue) and *runt* (red), 24 lbs. (H) *h3* (blue) and *runt* (red), 19 lbs. (I) *odr1* (blue) and *runt* (red), 24 lbs. (J) *wg* (red) and *en* (blue), 29 lbs. Panels are higher magnification DIC images of the white boxed areas in G, H, I and J. Coloured bars in panels are schematic representations of the accompanying stripes of gene expression, indicating stripe width and inter-stripe spacing. Stripe number as explained in Fig. 2. Embryos are flat-mounted preparations. Anterior is to the top. Scale bar is 100 μm.

**Fig. 2:**
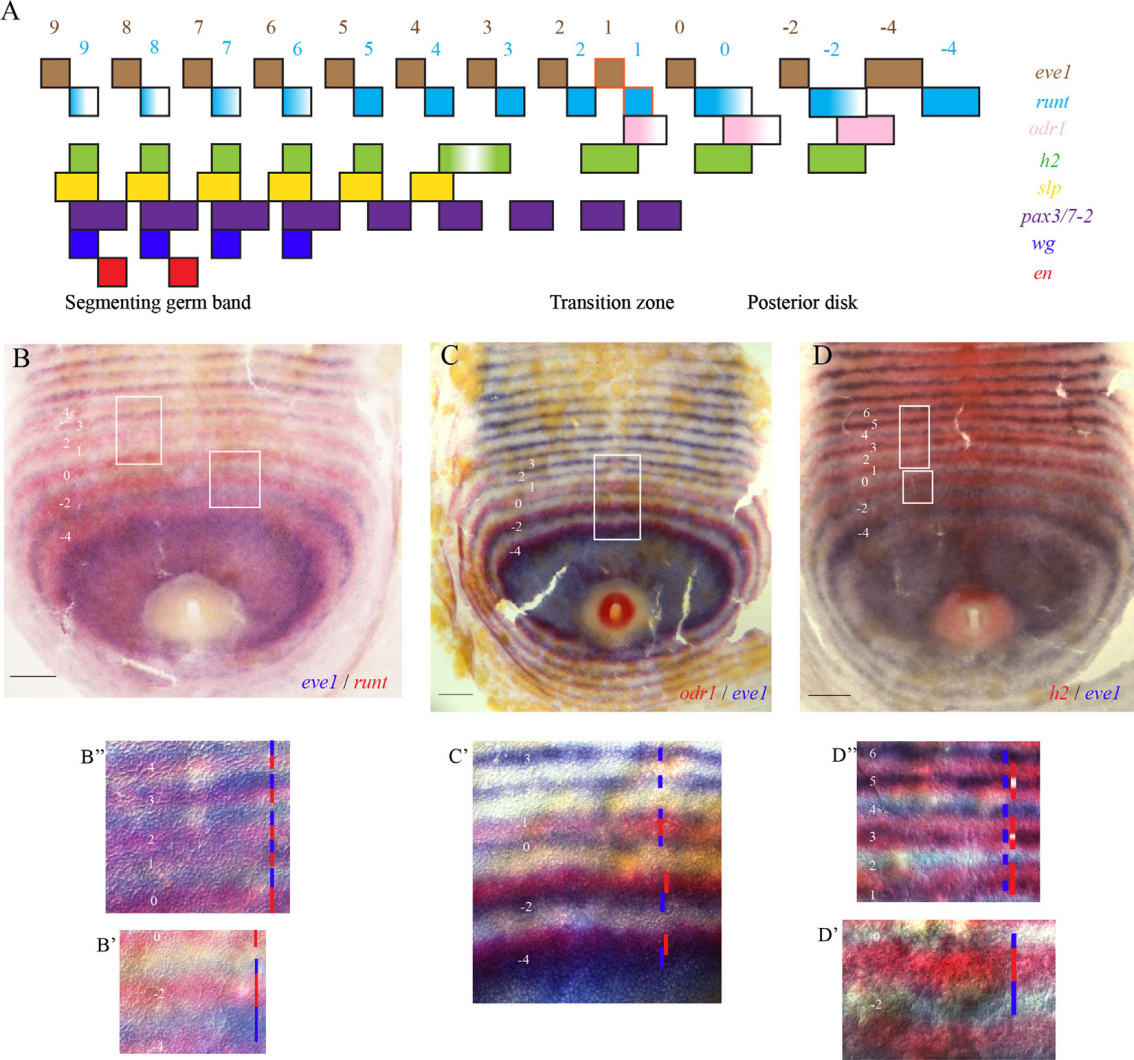
Schematic time series of expression of the *Strigamia* pair rule genes during the generation of periodic patterns. Panel A summarises the expression of pair rule and segment polarity genes during the progression of segment pattern formation in *Strigamia*. Each row represents the expression of a single gene along the anteroposterior axis from the posterior disc up to the specification of the first two parasegment boundaries. Anterior is to the left. Effectively, this presents a time series running from right to left. The relative horizontal position of domains summarises the degree of overlap, or otherwise, of the expression of the respective genes. For *eve1* and *runt*, an orange outline highlights the first appearance of stripes intercalating between the double segment stripes in the transition zone. Colour fading inside the boxes represents gradual clearing of the transcripts from cells. Brown and blue numbering indicates the relative position of *eve1* and *runt* stripes respectively. Stripe number 1 is the first intercalating stripe (see below). Panels B–D present examples of the data from which the map in panel A was generated. For a more complete dataset, see Supplementary Fig. 3. (B) Expression of *eve1* (blue) and *runt* (red) on a stage 4 embryo with 18 lbs. (C) Expression of *odr1* (red) and *eve1* (blue), 22 lbs. (D) Expression of *h2* (red) and *eve1* (blue), 17 lbs. Panels below are higher magnification DIC images of the white boxed areas in B, C, D. Coloured bars in panels are schematic representations of the accompanying stripes of gene expression, indicating stripe width and inter-stripe spacing. (B′) highlights boxed area on right hand side, (B″) on left hand side in B. (D′) highlights the lower, (D″) the upper boxed area in D. The stripe numbering indicates the relative position of a stripe in the pattern. The first intercalated stripe of expression is annotated as stripe 1. This is the first stripe that marks the transition to single segment periodicity. For stripes anterior to stripe 1, these are numbered in posterior-to-anterior progression 2, 3, 4 etc. The stripes posterior to stripe 1 are at double segment periodicity, and are thus numbered backwards in twos, in anterior-to-posterior order 0,−2, −4 etc. up to and including the first double segment ring which is clearly demarcated from the dynamic domain. Importantly, the numbering only pertains to *one* of the two genes shown on the double stain. In the bottom right-hand corner of each primary image, the two genes stained are indicated. The gene on the right-hand side of the separator defines the gene to which the stripe numbering annotated on the image applies. In all cases this gene is either *runt* or *eve1*. Anterior is to the top. Scale bar is 100 μm.

We have determined the registration of the primary and secondary pair rule genes, with respect to each other and to the segment polarity genes, using double *in situ* hybridisation. These data are summarised in an expression map (Fig. 2A, further supporting data in Fig. S3).

The map confirms previous work showing that there are two phases of periodic patterning – a double segment pre-pattern and the resolution of this to a single segment repeat. None of the genes that we have examined provide any evidence for single segment patterning occurring before cells enter the transition zone. Also, the summary shows that although the primary pair rule genes are expressed in similar patterns, they are expressed in different phases of the pattern repeat, and thus have distinct relationships with one another (Fig. 2A).

Fig. 2B, C, and D show three representative examples comparing the expression of *runt*, *odr1* and *h2* with *eve1*. For *eve1* and *runt*, at the anterior margin of the peri-proctodeal region, the dynamic pattern resolves into adjacent, space-filling stripes of *runt* and *eve1* that are at least three or four cell rows wide (Fig. 2B, B′). These stripes then narrow to around two cell rows, such that *runt* continues to abut *eve1* at its anterior margin, but an inter-stripe territory expressing neither gene appears posterior to the *runt* stripe, anterior to the next *eve1*-expressing cells. During the resolution to a single segment repeat, the intercalating stripes appear within this gap, and when fully resolved have the same relationship to one another as the stripes that derive from the narrowing of the primary stripes (Fig. 2B″).

For *odr1* and *eve1*, the pattern resolves initially into two overlapping stripes with a small anterior offset of *odr1* (Fig. 2C, C′). This pair of stripes is then separated from the next pair by non-expressing cells. Moving more anteriorly, the offset becomes more pronounced, such that they mature into largely non-overlapping stripes, with *odr1* abutting the anterior margin of *eve1*. In the transition zone, the intercalating stripe of *eve1* appears at the anterior border of the *odr1* stripe, and not overlapping with *odr1. odr1* is then inactivated.

For *h2* and *eve1*, during the double segment phase of the pattern, the expression dynamics are almost identical to those of *runt* and *eve1*, as described above (Fig. 2D, D′). In this phase, *h2* and *runt* are almost directly overlapping (Fig. S3G). However, *h2* has a different mode of transition to single segment periodicity. For *eve1* and *runt*, it seems that the intercalating stripes appear *de novo* in between the primary stripes (Fig. S2A, G). The *h2* primary stripes, on the other hand, appear to split by the repression of expression in cells within the stripe. The intercalating stripe of *eve1* is activated inside the *h2*-positive domain, approximately in the middle but with a posterior offset (Fig. 2D, D″). The *h2* transcripts are repressed in these *eve1*-positive cells. This repression leaves the anterior and posterior rows of *h2*-expressing cells intact, and separated from each other by non-expressing cells, thus producing segmental stripes.

A further major inference that can be drawn from the expression comparison is on the relative expression of different members of a gene family with respect to one another. *Strigamia* has three members each of the *eve* and *odr* gene families, and four members of the *hairy* family. We examined the expression of each gene with respect to a common reference gene, *runt* or *eve1*, in order to determine the registration of the family members to one another.

Each *eve* gene is activated early, in the peri-proctodeal region, but subsequently inactivated at a different position along the anteroposterior axis, so each is expressed in a distinct subset of the overall pattern. In each phase of the pattern, all three *eve* genes are expressed in the same spatial relationship with *runt* (Fig. S3A, B, C). Therefore, from the resolution of the first double segment ring, we can infer that all the *eve* family members are expressed in phase with one another. That is to say, in phases of the pattern where two or three of the genes are expressed, the transcripts are in the same cells and the stripes directly overlap.

The same logic can be applied to the *odr* family and the same conclusion drawn. Each *odr* gene is inactivated at a different anteroposterior position, but all three are expressed in the same spatial relationship with respect to *eve1* (Fig. S3D, E, F).

The situation is different for the *hairy* family. *h2* and *h3* have distinct spatial relationships with *runt*, and are therefore not expressed in phase with one another (Fig. S3G, H). Unfortunately, because the signal from the *h1* and *h4* probes is so weak, it has not been possible to obtain registration information from double stains with these probes.

Finally, the distinct expression domains of the Pax3/7 gene family warrant comment (Fig. S2L and P). The two members of this family in *Strigamia* are orthologous to three genes in *Drosophila* – *prd*, *gooseberry* and *gooseberry-neuro* (see above). *pax3/7-1* is activated later than *pax3/7-2* in the ectoderm, and only in medial cells that are likely to be neurectoderm. This neurectodermal expression of *pax3/7-1* resembles that of *gooseberry-neuro* in *Drosophila*, whereas the expression of *pax3/7-2* is most similar to the later segmental expression of *paired*, and of *gooseberry*. However, the one-to-one orthology of genes in this family remains uncertain.

### Conserved core of *pax3/7-2* and *slp* expression domains during parasegment boundary specification

Our observations support the hypothesis that the *en*/*wg* juxtaposition (i.e. the parasegment boundary) is the primary patterning boundary in each *Strigamia* segment, as it is in other arthropods. Fig. 2 shows that each new *en* stripe appears posteriorly adjacent to a *wg* stripe, and does not overlap with it. At the mid-stage in trunk segmentation depicted here, *en* expression is delayed with respect to *wg*, such that *wg* stripes appear two or three segments ahead of *en* (Fig. S3J). This temporal relationship changes at different stages of the segmentation process (data not shown). Even so, abutting stripes of *wg* and *en* are consistently established in each segment before any sign of morphological segmentation. When the segmental furrow forms, it lies immediately behind the *en* stripe. Later this furrow will carry the *en*-expressing cells down into it (Eibner, 2010) (L. Hayden and W. Arthur, in prep).

**Fig. 3:**
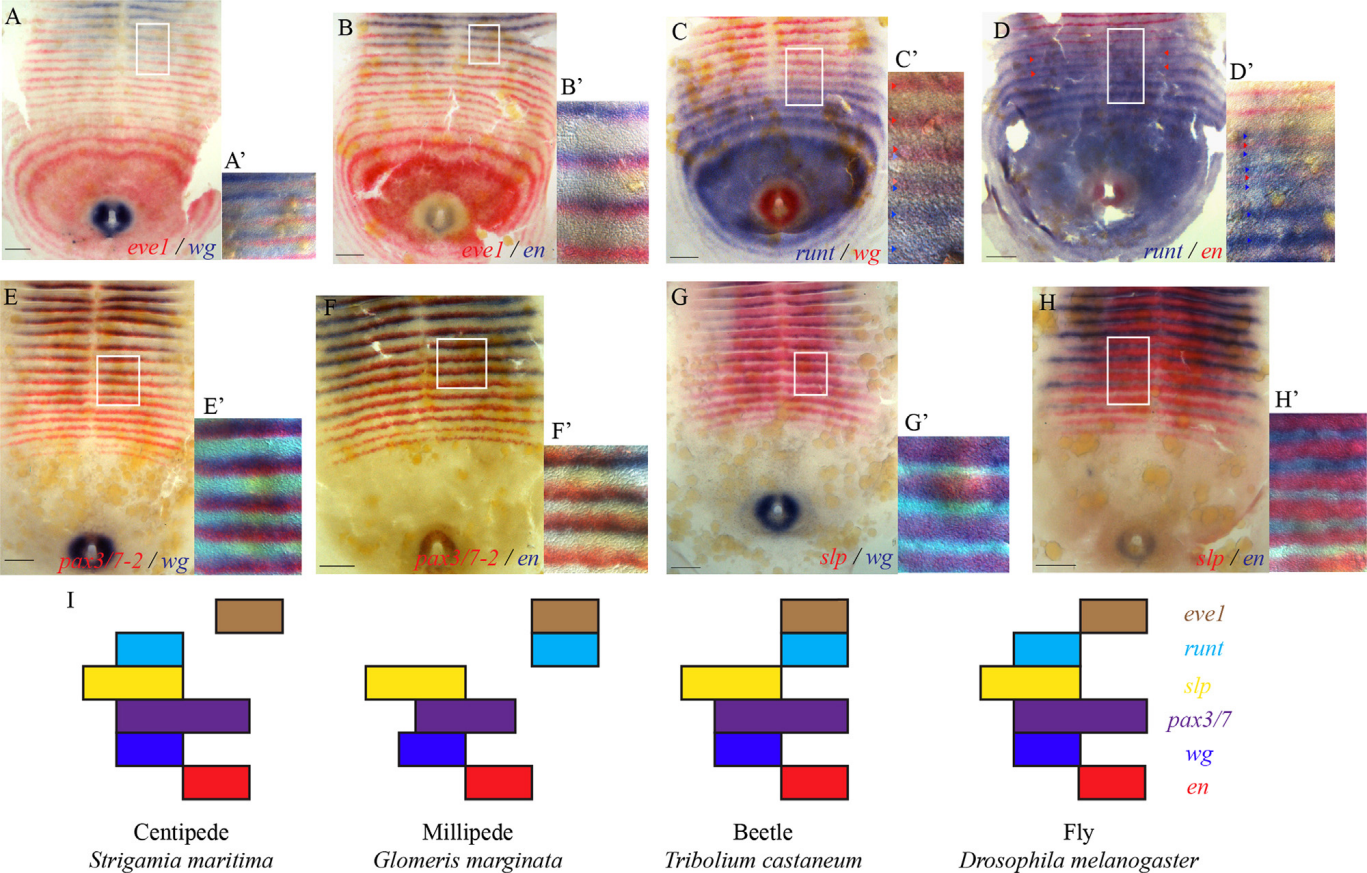
Conserved aspects of gene expression at the parasegment boundary. (A) Expression of *eve1* (red) and *wg* (blue) on a stage 4 embryo with 21 lbs. (A′) *eve1* and *wg* stripes are out of phase. (B) *eve1* (red) and *en* (blue), 21 lbs. (B′) *en* overlaps the anterior margin of *eve1*, but with an anterior offset. (C) *runt* (blue) and *wg* (red), 30 lbs. (C′) *wg* stripes appear directly overlapping *runt. wg* transcripts appear at the same time that *runt* transcripts are clearing from cells. (D) *runt* (blue) and *en* (red), 17 lbs. (D′) *runt* and *en* stripes are out of phase. (E) *pax3/7-2* (red) and *wg* (blue), 20 lbs. (E′) The first stripe of *wg* appears overlapping with the anterior one or two cell rows of the *pax3/7-2* stripe, and maintains this registration until morphological segmentation occurs. (F) *pax3/7-2* (red) and *en* (blue), 28 lbs. (F′) The first stripe of *en* appears overlapping with the posterior one or two cell rows of the *pax3/7-2* stripe. (G) *slp* (red) and *wg* (blue), 33 lbs. (G′) The first stripes of *wg* are overlapping with the stripes of *slp* expression. (H) *slp* (red) and *en* (blue), 29 lbs. (H′) Expression of *en* and *slp* are out of phase. Panels are higher magnification DIC images of the white boxed areas. Blue arrowheads in C′ and D′ indicate *runt* stripes, and red arrowheads indicate *wg* or *en* stripes respectively. Lighter colours in C′ indicate the gradual appearance of *wg* transcripts at the same time that *runt* transcripts are clearing. Embryos are flat-mounted preparations. Anterior is to the top. Scale bar is 100 μm. (I) Schematic comparison of the relationships of expression domains of key genes at the parasegment boundary between insects and myriapods. In *Drosophila* and *Tribolium*, the mechanism of *wg* and *en* regulation is different in alternate parasegments, and the relationship of expression domains of some genes is also different in alternate parasegments. For *Drosophila* the summary of relative gene expression is shown for the parasegment boundaries between odd-numbered *en* stripes and even-numbered *wg* stripes. But for *Tribolium*, the expression data is shown for the opposite parasegment registration, that is to say, even-numbered *en* and odd-numbered *wg* stripes. Anterior is to the left. Modified from Janssen et al., 2011.

Fig. 3 compares the relative expression domains of some key genes at the point of parasegment boundary specification in *Strigamia* with the data available in other arthropods. This comparison shows that the expression domains of the secondary pair rule genes *slp* and *pax3/7-2* have a conserved registration with the domains of *wg* and *en* at the parasegment boundary.

Each new stripe of *wg* appears overlapping with the anterior one or two cell rows of a *pax3/7-2* stripe, and maintains this registration until morphological segmentation occurs (Fig. 3E, E′). Conversely, new stripes of *en* appear overlapping with the posterior one or two cell rows of the *pax3/7-2* stripe, and similarly maintain this registration (Fig. 3F, F′). For technical reasons it has not been possible to monitor the expression of all three genes simultaneously, but it is clear that *pax3/7-2* is co-expressed in some, if not all, of both the *wg*- and *en*-positive cells, and thus overlaps the parasegment boundary.

Each new stripe of *wg* also overlaps with a stripe of *slp* expression (Fig. 3G, G′). Within the limitations of double colorimetric *in situ*, it appears that all *wg*-positive cells are encompassed within the *slp* domain. In contrast, the expression of *en* and *slp* appear to be out of phase, with no or very little overlap (Fig. 3H, H′).

These observations confirm the conservation of expression of the key secondary pair rule genes at the parasegment boundary across arthropods (Fig. 3I).

### Evolutionary flexibility of expression of primary pair rule genes

The *eve*, *runt* and *hairy* homologues show a greater degree of divergence in the spatial relationships of their expression than the secondary pair rule or segment polarity gene homologues (Fig. 3).

Examining the registration of *eve1* at the parasegment boundary, we observe that the *wg* stripes are out-of-phase with the *eve1* stripes (Fig. 3A, A′). The appearance of the first *en* stripe is partially overlapping with the anterior margin of the *eve1* stripe (Fig. 3B, B′). It appears that the *en* stripe is anteriorly offset, such that some *en*-expressing cells are outside of the *eve* domain. This spatial relationship between *eve1* and *en* differs from that in both *Glomeris*, where there is no overlap, and in *Drosophila* and *Tribolium*, where the *en* stripe is entirely within the *eve* domain (Fig. 3I) (Janssen et al., 2011; Lawrence et al., 1987; Patel et al., 1994).

For *runt*, the first stripes of *en* and *wg* initiate in a region of the germ band where *runt* transcripts are beginning to clear from cells (Fig. 3C, D). The first stripes of *wg* expression initiate directly on top of the *runt* stripes (Fig. 3C, C′). The first stripes of *en* appear in between the segmental stripes of *runt*, and do not overlap (Fig. 3D, D′). At the mid-segmentation stage examined here, soon after the activation of two stripes of *wg* or *en*, *runt* transcripts have completely disappeared from this region of the germ band. Comparing this pattern with other arthropods (Fig. 3I), the spatial relationship of *runt* with respect to *eve* and to the parasegment boundary has diverged between flies and beetles, and between centipedes and millipedes.

Furthermore, in the double segment phase of segment patterning, the registration of the *runt* homologue and at least two of the *hairy* homologues has diverged between fly and centipede. In *Drosophila*, the double segment stripes of *hairy* and *runt* are expressed out-of-phase. In the double segment phase of *Strigamia* however, *runt* and *h2* are expressed in phase; and *runt* and *h3* stripes are partially overlapping (Fig. S3G, H). Overall, these observations confirm earlier suggestions from other arthropods that the upstream tiers of segment patterning are more evolutionarily flexible (Peel et al., 2005).

### Periodic expression of *Strigamia* pair rule genes is restricted to the ectoderm

**Fig. 4:**
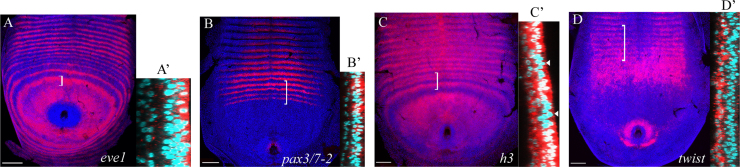
Expression of pair rule genes in the ectoderm and mesoderm. In all panels, the side panels show a higher magnification, orthogonal section along the anteroposterior axis of the bracketed area in the main panel. (A) Expression of *eve1* in the posterior disc and germ band of a stage 4 embryo with 20 leg-bearing segments. (A′) *eve1* expression is restricted to the ectoderm. Transcripts appear to be predominantly apically localised. (B) *pax3/7-2* expression, 28 lbs. (B′) *pax3/7-2* expression is restricted to the ectoderm. (C) *h3* expression, 23 lbs. (C′) The striped expression of *h3* is restricted to the ectoderm. White arrowheads indicate the ectodermal inter-stripe regions. *h3* has an additional uniform expression domain in the mesoderm. (D) *twist* expression, 18 lbs. (D′) *twist* is restricted to the mesoderm. The *twist*-positive cells line up into rows and arrange themselves underneath the segmented ectoderm. In the overview and accompanying cross-section images, the RNA is stained in red and shown overlaying the nuclear stain. In the overview images, nuclei are shown in dark blue. In the cross-section images the nuclei are shown in cyan, which gives a clearer distinction of the two germ layers and the expression of the RNA in relation to them. Embryos are flat-mounted preparations. Anterior is to the top. In cross-sections, the ectoderm is oriented on the right, and the underlying mesodermal cells to the left. Scale bar is 100 μm (overviews only).

From an analysis of nuclear-stained embryos in cross-section, it is clear that there are two distinct cell layers in the posterior disc (Fig. 4). It is most likely that the surface layer is the ectoderm, and the deep layer is the mesoderm. To test this, we examined the expression of the *Strigamia* homologue of the mesodermal marker gene *twist* (Fig. 4D). At the stage examined, the surface cells never express *twist*, consistent with an ectodermal identity. Except in the immediate vicinity of the proctodeum itself, the deep cells posterior to the transition zone are also *twist-*negative. However, deep cells in the transition zone do express *twist*. These *twist*-expressing cells are initially loosely organised, but more anteriorly they line up into rows that come to lie directly underneath the ectodermal segments. More anteriorly still, these cells organise themselves into coelomic sacs. The *twist*-negative deep cells of the posterior disc are continuous with the *twist*-positive cells that will form the mesoderm in the segmented germ band. Therefore on the basis of the behaviour and topology of the cells, as well as the expression of a mesodermal marker gene, we conclude that these cells are indeed mesodermal precursors.

**Supplementary Fig. S4:**
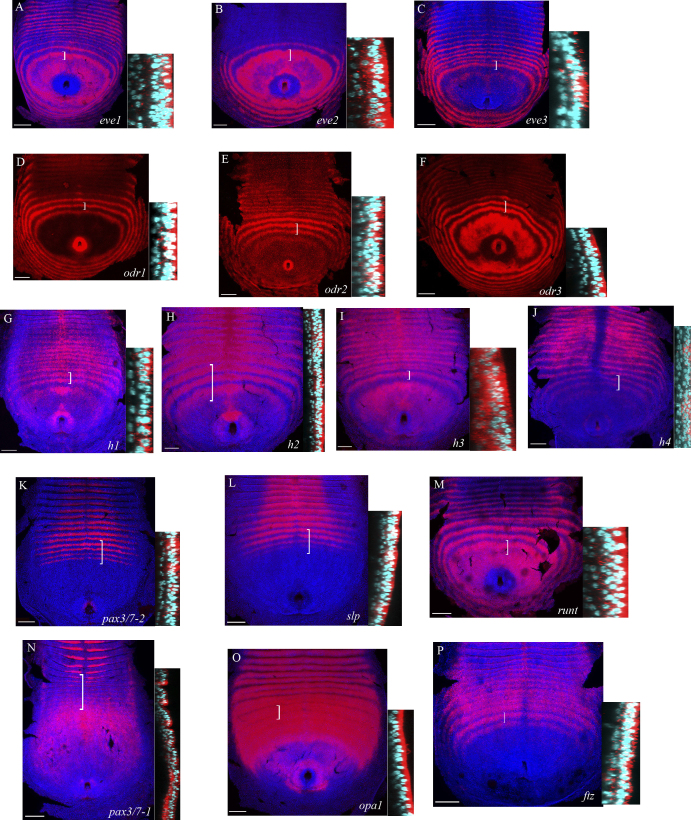
Expression of pair rule genes in different germ layers. (A) Expression of *eve1*, 17 lbs. (B) *eve2*, 17 lbs. (C) *eve3*, 21 lbs. (D) *odr1*, 25 lbs. (E) *odr2*, 18 lbs. (F) *odr3*, 18 lbs. (G) *h1*, 14 lbs. (H) *h2*, 28 lbs. (I) *h3*, 23 lbs. (J) *h4*, 16 lbs. (K) *pax3/7-2*, 28 lbs. (L) *slp*, 33 lbs. (M) *runt*, 20 lbs. (N) *pax3/7-1*, 22 lbs. (O) *opa1*, 25 lbs. (P) *ftz*, 18 lbs. The side panels show a higher magnification, orthogonal section along the anteroposterior axis of the bracketed area in the main panel. In the overview and accompanying cross-section images, the RNA is stained in red and shown overlaying the nuclear stain. In the overview images, nuclei are shown in dark blue. In the cross-section images the nuclei are shown in cyan, which gives a clearer distinction of the two germ layers and the expression of the RNA in relation to them. Embryos are flat-mounted preparations. Anterior is to the top. In cross-sections, the ectoderm is oriented on the right, and the underlying mesodermal cells to the left. Scale bar is 100 μm (overviews only).

For all the pair rule gene homologues, the expression of the genes in dynamic and periodic patterns, in the posterior disc and transition zone, is restricted to the ectodermal cell layer (Figs. 4A, B and S4). Labelling of the mesoderm is much weaker, probably at background levels, and this labelling is not modulated into double or single segment patterns. This suggests that any direct role of these genes in segment pattern formation is restricted to the ectoderm.

There are two genes that have additional domains of expression in the mesoderm, *h3* and *pax3/7-1* (Figs. 4C and S4N). *h3* is expressed uniformly throughout the mesodermal layer of the posterior disc and continues into the mesoderm of the morphological segments. However, the dynamic and striped domains of expression are restricted to the ectoderm (Fig. 4C). *pax3/7-1* is never expressed in the ectoderm before the appearance of morphological segments. It is expressed in mesodermal precursor cells throughout much of the posterior disc (Fig. S4N). As furrows form to demarcate the segments, *pax3/7-1* is inactivated in the mesodermal cells, and reinitiates in a specific row of ectodermal cells at the posterior of each segment. This expression is restricted to the ventral neurectoderm territory. This pattern is distinct from that of all other pair rule genes. Importantly, when viewed in XZ or YZ cross-sections, the mesodermal expression of both *h3* and *pax3/7-1* shows *no* evidence of modulation into double or single segment patterns. Expression appears to be uniform in intensity. Any appearance of segmental modulation in surface views (XY) is actually caused by cell rearrangements, as the ectodermal furrows fold inwards and the mesodermal cells organise into rows.

In summary, the data support the hypothesis that any direct role of the pair rule gene homologues in the generation of periodic patterning is restricted to the ectoderm. This implies that mesoderm segmentation is either induced by signals from the overlying ectoderm, or occurs through an independent developmental mechanism.

## Discussion

We have shown that, with the exception of *pax3/7-1* and *opa2*, all *Strigamia* homologues of the *Drosophila* pair rule genes are expressed in the ectodermal cell layer of the posterior disc and/or forming germ band before the formation of morphological segments. Most are expressed in periodic patterns and their expression precedes the activation of the segment polarity genes *wg* and *en*, consistent with a role in the generation of segmental pattern.

It might be argued that most genes would show such periodic patterns in the posterior disc and transition zone, in response to a much smaller subset of transcription factors that are actually necessary for segment patterning. To assess this argument, we examined the expression of a sample of 25 other *Strigamia* genes that we and our colleagues had cloned as mesodermal or neural markers for use in other studies, and which were not known to be involved in arthropod trunk segmentation from studies in other arthropods. None of these 25 is expressed in a dynamic pattern in the peri-proctodeal region; and the majority (18/25) is not expressed before the appearance of morphological segments (data not shown). Therefore, the expression dynamics of the *Strigamia* pair rule gene set is distinctive, providing suggestive evidence that many if not most of these genes play a role in segmentation.

As we have no tools for gene manipulation in *Strigamia*, these expression data provide our best proxy for inferring gene function. However, we must be cautious. The patterned expression of a gene during a process does not mean that it plays any necessary role in that process. For example, the striped expression of *ftz* and *hairy* in *Tribolium* is consistent with a function in segmentation, but it is known that RNAi knockdown of these genes gives no detectable trunk segmentation phenotype (Aranda et al., 2008; Choe et al., 2006). In these cases, regulatory linkage into the segmentation gene network might reflect a retained vestige of an ancestral function that is no longer necessary, or a role in coupling the segmentation network to other processes (e.g. possibly Hox gene regulation, in the case of *ftz*). Such patterns may even have arisen by chance, persisting because they are not deleterious. That being said, we interpret our data as suggesting that the set of transcription factors encoded by the pair rule gene homologues has been involved in segment pattern generation since at least the origin of the major living arthropod lineages.

### Conservation of hierarchical network structure

In the two major insect models *Tribolium* and *Drosophila*, in at least one representative of the chelicerates, the spider *Cupiennius salei*, and now in two myriapods, the millipede *Glomeris marginata* and the centipede *Strigamia maritima,* there is evidence for a hierarchical organisation of the pair rule genes (Choe et al., 2006; Damen et al., 2000, 2005; Ingham and Gergen, 1988; Ingham, 1988; Janssen et al., 2011; Schroeder et al., 2004). In particular, *eve* and *runt* homologues are identified as primary pair rule genes, expressed early and where tested functioning upstream; *slp* and *pax3/7* homologues as secondary pair rule genes, expressed later and functioning downstream, in all species examined. This feature of the network organisation may be more stable than the exact regulatory linkages between individual genes, which have clearly diverged in different insect lineages – and of which we as yet know essentially nothing in non-insect arthropods.

### An ancestral arthropod patterning system for specifying the parasegment boundary

In both insects and myriapods, a *pax3/7* homologue is expressed across the parasegment boundary in both *wg*- and *en*-positive cells; and a *slp* homologue is expressed only in *wg*-positive cells (Choe and Brown, 2009; Janssen et al., 2011). Therefore in *Strigamia pax3/7* and *slp* homologues are expressed in the right cells and at the right time to have a function in regulating *wg* and *en* during parasegment boundary specification, but this remains to be tested.

Within the insects, the functional data indicates a mixed picture. RNAi experiments in *Tribolium* confirm that these genes have a role in segment patterning, and are required for the correct establishment of parasegmental stripes of *wg* and *en* (Choe and Brown, 2007). However, the regulatory interactions between genes that underlie the execution of this patterning function have diverged from *Drosophila* (Choe and Brown, 2009). This is consistent with the growing evidence that developmental systems drift is pervasive – the same or similar phenotypic results are frequently accomplished through alternative developmental pathways (True and Haag, 2001).

Given our data in *Strigamia*, we propose that the involvement of *slp* and *prd* in a regulatory module that sets the borders of *wg* and *en* stripes is a stable and ancient feature of segment patterning, but that the involvement of other genes and the exact linkages between genes in the module is more evolutionarily labile, and will have diverged in different lineages. It is important to study these questions in other non-insect arthropods, particularly crustaceans and chelicerates, in order to build a more complete catalogue of those parts of the segmentation gene regulatory network which are stable, and those which are more flexible. This comparative dataset will be very useful for testing hypotheses on how the location and connectedness of regulatory modules in a network affect its susceptibility to evolutionary change.

### Evolutionary flexibility of primary pair rule genes

In contrast to the high degree of conservation of the secondary pair rule and segment polarity genes at the parasegment boundary, the expression of the primary pair rule genes, during double segment patterning and at the parasegment boundary, appears to be more evolutionarily flexible (Figs. 2 and 3).

The expression of *eve1* shows a mixture of conserved and divergent aspects. At the parasegment boundary, *eve1* is expressed out-of-phase with *wg* stripes, and partially overlapping with *en* stripes at its anterior margin (Fig. 3A, B). The observation that the overlap between the anterior margin of *eve1* and the first *en* stripe is only partial is significant. This is because, assuming there are no differences in expression between *eve* transcripts and protein, the presence of *en*-expressing cells outside of the *eve1* domain suggests that *eve1* cannot be responsible for regulating the entire *en* stripe. This is different from the situation found in *Drosophila* and *Tribolium*, where all *en*-positive cells are encompassed within the *eve* stripe (Lawrence et al., 1987; Patel et al., 1994), but more consistent with the pattern described in *Glomeris*, where all *en*-positive cells are outside the *eve* domain (Janssen et al., 2011). It suggests that the role of *eve* in setting the borders of the *en* stripes might have diverged or been lost in myriapods.

In comparison with other arthropods, the expression of *runt* with respect to the other examined genes at the parasegment boundary is particularly flexible. Its relative expression domain is divergent between flies and beetles, and between millipedes and centipedes (Choe and Brown, 2009; Janssen et al., 2011). During double segment patterning, the registration of *runt* with respect to two of the *hairy* homologues is also divergent between *Strigamia* and *Drosophila* (Fig. S3G, H). This evolutionary flexibility in spatial registration might reflect corresponding differences in its functional relationship with other pair rule genes.

It is known from *Drosophila* and mammalian systems that *runt* exhibits context-dependent transcriptional regulation (Collins et al., 2009; Swantek and Gergen, 2004; Walrad et al., 2011). It can act as a transcriptional repressor or activator, depending on its interactions with different cofactors. Given the deep conservation of the Runx family of transcription factors across metazoans (Sullivan et al., 2008), it is highly likely that this biochemical potential of *runt* is also conserved in non-insect arthropods. On this basis, one possible hypothesis for the flexibility of *runt* is that its dual regulatory properties enable it to occupy many different positions in the transcriptional network. Also, the biochemical simplicity of switching between being an activator and repressor through an exchange of cofactors may have facilitated its fast evolution.

### Restriction of dynamic and periodic expression to the ectoderm

None of the pair rule gene homologues that we have examined is expressed in a dynamic or periodic pattern in the mesoderm. With the exception of *pax3/7-1* and *h3*, which are expressed in non-periodic mesodermal domains, the rest of the genes are either never expressed in the mesodermal cells of the posterior disc and transition zone, or expressed at such low levels as to be indistinguishable from background.

In *Drosophila*, pair rule genes are expressed in the mesoderm, and it is known that periodic stripes of *eve* and *slp* are necessary for correct patterning of the mesoderm into cardiac, somatic and visceral fates (Azpiazu et al., 1996; Riechmann et al., 1997). On the other hand, most of *Drosophila* segmentation is complete before gastrulation, and it is known that this is a derived condition within the arthropods. *Tribolium* is more typical for the arthropods, where segments are generated after gastrulation, in a sequential manner from a growth zone with both ectodermal and mesodermal progenitor cells. In *Tribolium*, *eve* and *odd* homologues are expressed in the mesoderm of the growth zone before morphological segmentation (Sarrazin et al., 2012), but their function in these cells is unknown.

In general mesoderm segmentation is not understood in *Tribolium*, but there are a couple of suggestive observations. First, the re-activation of *twist* in segmental bands in the growth zone mesoderm occurs just anterior to the appearance of newly formed *en* stripes – and thus in a region where *wg* and *hedgehog* are being transcribed, and might have begun signalling to surrounding cells (Handel et al., 2005). However, it is not known whether this temporal correlation reflects a causal link between ectodermal signalling and mesoderm segmentation. Second, it is known that in a dorsalised *Tribolium* embryo with no mesoderm, ectoderm segmentation still occurs (da Fonseca et al., 2008). This supports the hypothesis that ectoderm segmentation is autonomous in *Tribolium*.

There are no genes examined to date in *Strigamia* that are expressed in a periodic pattern in the mesoderm before such patterns are established in the ectoderm. If this holds true as more genes are studied, it is suggestive evidence that mesoderm segmentation might be induced by signals from the overlying ectoderm. There is supporting evidence from *Drosophila* and the amphipod crustacean *Parhyale hawaiensis* that mesoderm segmentation is secondary and at least partially dependent on the ectoderm (Azpiazu et al., 1996; Frasch, 1999; Hannibal et al., 2012). In all it suggests that primary segmentation of the ectoderm may be the ancestral arthropod condition. It is an interesting area for future work to investigate how mesoderm segmentation occurs in other sequentially segmenting arthropods.

## Footnotes

Appendix A Supplementary data associated with this article can be found in the online version at 10.1016/j.ydbio.2013.06.017.

## References

[bib1] Akam M. (1987). The molecular basis for metameric pattern in the *Drosophila* embryo. Development.

[bib2] Aranda M., Marques-Souza H., Bayer T., Tautz D. (2008). The role of the segmentation gene *hairy* in *Tribolium*. Dev. Genes Evol..

[bib3] Azpiazu N., Lawrence P.A., Vincent J.P., Frasch M. (1996). Segmentation and specification of the *Drosophila* mesoderm. Genes Dev..

[bib4] Balczarek K.A., Lai Z.C., Kumar S. (1997). Evolution and functional diversification of the paired box (Pax) DNA-binding domains. Mol. Biol. Evol..

[bib5] Bao R., Friedrich M. (2008). Conserved cluster organization of insect Runx genes. Dev. Genes Evol..

[bib6] Brena C., Akam M. (2012). The embryonic development of the centipede *Strigamia maritima*. Dev. Biol..

[bib7] Chipman A.D., Akam M. (2008). The segmentation cascade in the centipede *Strigamia maritima*: Involvement of the Notch pathway and pair-rule gene homologues. Dev. Biol..

[bib8] Chipman A.D., Arthur W., Akam M. (2004). A double segment periodicity underlies segment generation in centipede development. Curr. Biol..

[bib9] Chipman A.D., Arthur W., Akam M. (2004). Early development and segment formation in the centipede, *Strigamia maritima* (Geophilomorpha). Evol. Dev..

[bib10] Chipman A.D., Stollewerk A. (2006). Specification of neural precursor identity in the geophilomorph centipede *Strigamia maritima*. Dev. Biol..

[bib11] Choe C.P., Brown S.J. (2007). Evolutionary flexibility of pair-rule patterning revealed by functional analysis of secondary pair-rule genes, *paired* and *sloppy-paired* in the short-germ insect, *Tribolium castaneum*. Dev. Biol..

[bib12] Choe C.P., Brown S.J. (2009). Genetic regulation of *engrailed* and *wingless* in *Tribolium* segmentation and the evolution of pair-rule segmentation. Dev. Biol..

[bib13] Choe C.P., Miller S.C., Brown S.J. (2006). A pair-rule gene circuit defines segments sequentially in the short-germ insect *Tribolium castaneum*. Proc. Natl. Acad. Sci. USA.

[bib14] Collins A., Littman D.R., Taniuchi I. (2009). RUNX proteins in transcription factor networks that regulate T-cell lineage choice. Nat. Rev. Immunol..

[bib15] da Fonseca R.N., von Levetzow C., Kaischeuer P., Basal A., van der Zee M., Roth S. (2008). Self-regulatory circuits in dorsoventral axis formation of the short-germ beetle *Tribolium castaneum*. Dev. Cell..

[bib16] Damen W.G.M. (2007). Evolutionary conservation and divergence of the segmentation process in arthropods. Dev. Dyn..

[bib17] Damen W.G.M., Janssen R., Prpic N.M. (2005). Pair rule gene orthologs in spider segmentation. Evol. Dev..

[bib18] Damen W.G.M., Weller M., Tautz D. (2000). Expression patterns of *hairy*, *even-skipped*, and *runt* in the spider *Cupiennius salei* imply that these genes were segmentation genes in a basal arthropod. Proc. Natl. Acad. Sci. USA.

[bib19] Davis G.K., Jaramillo C.A., Patel N.H. (2001). Pax group III genes and the evolution of insect pair-rule patterning. Development.

[bib20] Edgar R.C. (2004). MUSCLE: multiple sequence alignment with high accuracy and high throughput. Nucleic Acids Res..

[bib21] Eibner, C., 2010. Segment formation in the centipede *Strigamia maritima* with emphasis on segment polarity genes and environmental factors (doctoral thesis). National University of Ireland Galway, Galway.

[bib22] Farzana L., Brown S.J. (2008). *Hedgehog* signaling pathway function conserved in *Tribolium* segmentation. Dev. Genes Evol..

[bib23] Frasch M. (1999). Intersecting signalling and transcriptional pathways in *Drosophila* heart specification. Semin. Cell Dev. Biol..

[bib24] Guindon S., Gascuel O. (2003). A simple, fast, and accurate algorithm to estimate large phylogenies by maximum likelihood. Syst. Biol..

[bib25] Handel K., Basal A., Fan X., Roth S. (2005). *Tribolium castaneum twist*: gastrulation and mesoderm formation in a short-germ beetle. Dev. Genes Evol..

[bib26] Hannibal R.L., Price A.L., Patel N.H. (2012). The functional relationship between ectodermal and mesodermal segmentation in the crustacean, *Parhyale hawaiensis*. Dev. Biol..

[bib27] Hodcroft, E., 2012. TreeCollapserCL 〈http://emmahodcroft.com/TreeCollapseCL3.html〉

[bib28] Ingham P., Gergen P. (1988). Interactions between the pair-rule genes *runt*, *hairy*, *even-skipped* and *fushi-tarazu* and the establishment of periodic pattern in the *Drosophila* embryo. Development.

[bib29] Ingham P.W. (1988). The molecular genetics of embryonic pattern formation in *Drosophila*. Nature.

[bib30] Jaeger J., Surkova S., Blagov M., Janssens H., Kosman D., Kozlov K.N., Manu, Myasnikova E., Vanario-Alonso C.E., Samsonova M., Sharp D.H., Reinitz J. (2004). Dynamic control of positional information in the early *Drosophila* embryo. Nature.

[bib31] Janssen R., Budd G.E., Prpic N.-M., Damen W.G. (2011). Expression of myriapod pair rule gene orthologs. EvoDevo.

[bib32] Kettle C., Johnstone J., Jowett T., Arthur H., Arthur W. (2003). The pattern of segment formation, as revealed by *engrailed* expression, in a centipede with a variable number of segments. Evol. Dev..

[bib33] Lawrence P.A., Johnston P., Macdonald P., Struhl G. (1987). Borders of parasegments in *Drosophila* embryos are delimited by the *fushi-tarazu* and *even-skipped* genes. Nature.

[bib34] Lee H.H., Frasch M. (2004). Survey of forkhead domain encoding genes in the *Drosophila* genome: classification and embryonic expression patterns. Dev. Dyn..

[bib35] Ludwig M.Z., Bergman C., Patel N.H., Kreitman M. (2000). Evidence for stabilizing selection in a eukaryotic enhancer element. Nature.

[bib36] Mazet F., Yu J.K., Liberles D.A., Holland L.Z., Shimeld S.M. (2003). Phylogenetic relationships of the Fox (Forkhead) gene family in the Bilateria. Gene.

[bib37] Nusslein-Volhard C., Wieschaus E. (1980). Mutations affecting segment number and polarity in *Drosophila*. Nature.

[bib38] Pankratz M.J., Jaeckle H., Bate M., Martinez Arias A. (1993). Blastoderm segmentation. The Development of *Drosophila melanogaster*.

[bib39] Patel N.H., Condron B.G., Zinn K. (1994). Pair-rule expression patterns of *even-skipped* are found in both short-germ and long-germ beetles. Nature.

[bib40] Peel A.D., Chipman A.D., Akam M. (2005). Arthropod segmentation: beyond the *Drosophila* paradigm. Nat. Rev. Genet..

[bib41] Rambaut, A., 2009. FigTree 〈http://tree.bio.ed.ac.uk/software/figtree/〉.

[bib42] Riechmann V., Irion U., Wilson R., Grosskortenhaus R., Leptin M. (1997). Control of cell fates and segmentation in the *Drosophila* mesoderm. Development.

[bib43] Romano L.A., Wray G.A. (2003). Conservation of *Endo16* expression in sea urchins despite evolutionary divergence in both cis and trans-acting components of transcriptional regulation. Development.

[bib44] Sanchez R., Serra F., Tarraga J., Medina I., Carbonell J., Pulido L., de Maria A., Capella-Gutierrez S., Huerta-Cepas J., Gabaldon T., Dopazo J., Dopazo H. (2011). Phylemon 2.0: a suite of web-tools for molecular evolution, phylogenetics, phylogenomics and hypotheses testing. Nucleic Acids Res..

[bib45] Sarrazin A.F., Peel A.D., Averof M. (2012). A segmentation clock with two-segment periodicity in insects. Science.

[bib46] Schroeder M.D., Greer C., Gaul U. (2011). How to make stripes: deciphering the transition from non-periodic to periodic patterns in *Drosophila* segmentation. Development.

[bib47] Schroeder M.D., Pearce M., Fak J., Fan H.Q., Unnerstall U., Emberly E., Rajewsky N., Siggia E.D., Gaul U. (2004). Transcriptional control in the segmentation gene network of *Drosophila*. PLoS Biol..

[bib48] Sullivan J.C., Sher D., Eisenstein M., Shigesada K., Reitzel A.M., Marlow H., Levanon D., Groner Y., Finnerty J.R., Gat U. (2008). The evolutionary origin of the Runx/CBFbeta transcription factors—studies of the most basal metazoans. BMC Evol. Biol..

[bib49] Swantek D., Gergen J.P. (2004). *Ftz* modulates *Runt*-dependent activation and repression of segment-polarity gene transcription. Development.

[bib50] True J.R., Haag E.S. (2001). Developmental system drift and flexibility in evolutionary trajectories. Evol. Dev..

[bib51] Walrad P.B., Hang S., Gergen J.P. (2011). *Hairless* is a cofactor for *Runt*-dependent transcriptional regulation. Mol. Biol. Cell..

[bib52] Wilson M.J., Dearden P.K. (2012). Pair-rule gene orthologues have unexpected maternal roles in the honeybee (*Apis mellifera*). Plos One.

